# Indication for Co-evolution of *Lactobacillus johnsonii* with its hosts

**DOI:** 10.1186/1471-2180-12-149

**Published:** 2012-07-25

**Authors:** Keren Buhnik-Rosenblau, Vera Matsko-Efimov, Minju Jung, Heuynkil Shin, Yael Danin-Poleg, Yechezkel Kashi

**Affiliations:** 1 Department of Biotechnology and Food Engineering, Technion-Israel Institute of Technology, Haifa, Israel; 2 School of Life Sciences, Handong Global University, Pohang, Gyungbuk, 791-708, Korea

## Abstract

**Background:**

The intestinal microbiota, composed of complex bacterial populations, is host-specific and affected by environmental factors as well as host genetics. One important bacterial group is the lactic acid bacteria (LAB), which include many health-promoting strains. Here, we studied the genetic variation within a potentially probiotic LAB species, *Lactobacillus johnsonii*, isolated from various hosts.

**Results:**

A wide survey of 104 fecal samples was carried out for the isolation of *L. johnsonii*. As part of the isolation procedure, terminal restriction fragment length polymorphism (tRFLP) was performed to identify *L. johnsonii* within a selected narrow spectrum of fecal LAB. The tRFLP results showed host specificity of two bacterial species, the *Enterococcus faecium* species cluster and *Lactobacillus intestinalis*, to different host taxonomic groups while the appearance of *L. johnsonii* and *E. faecalis* was not correlated with any taxonomic group. The survey ultimately resulted in the isolation of *L. johnsonii* from few host species. The genetic variation among the 47 *L. johnsonii* strains isolated from the various hosts was analyzed based on variation at simple sequence repeats (SSR) loci and multi-locus sequence typing (MLST) of conserved hypothetical genes. The genetic relationships among the strains inferred by each of the methods were similar, revealing three different clusters of *L. johnsonii* strains, each cluster consisting of strains from a different host, *i.e.* chickens, humans or mice.

**Conclusions:**

Our typing results support phylogenetic separation of *L. johnsonii* strains isolated from different animal hosts, suggesting specificity of *L. johnsonii* strains to their hosts. Taken together with the tRFLP results, that indicated the association of specific LAB species with the host taxonomy, our study supports co-evolution of the host and its intestinal lactic acid bacteria.

## Background

The intestinal microbiota consists of hundreds to thousands of bacterial species which play an important role in normal gut functioning and are crucial for maintaining the organism in good health. It is composed of complex bacterial populations that have recently been found to be host-specific [1-3], a result of variations in environmental factors [4-6] and host genetics [7-11].

One important group of bacteria colonizing the gut is the lactic acid bacteria (LAB), a heterogeneous group of gram-positive rods and cocci that belong to the phylum *Firmicutes*. There are indications of a correlation between oral administration of some LAB strains and improvement of gut health disorders, such as pouchitis, ulcerative colitis, infectious diarrhea, antibiotic-associated diarrhea, traveler’s diarrhea, necrotizing enterocolitis, atopic eczema and *Helicobacter pylori* infections [12-16]. The largest bacterial genus in the LAB is *Lactobacillus*. It is highly diverged and consists of over a hundred species [17,18]. Lactobacilli are widely used in food fermentation and are well known for their preservative ability as well as for their positive contribution to texture and flavor formation in many food products. In addition, several well-characterized probiotic strains (live microorganisms which, when administered in adequate amounts, confer a health benefit on the host; FAO/WHO Guidelines, 2002, ftp://ftp.fao.org/es/esn/food/wgreport2.pdf) belonging to this genus are used by the food and pharmaceutical industries, and new probiotic lactobacilli strains are discovered. One of the most intensively investigated *Lactobacillus* species is *Lactobacillus johnsonii*, which has been reported so far to inhabit the gastrointestinal tracts (GITs) of several hosts, including humans, mice, dogs, poultry, pigs and honeybees [19-23]. Specific *L. johnsonii* strains are known for their probiotic activities [24-28] and some, such as *L. johnsonii* NCC 533 [29], are also used by the industry. Probiotic characteristics are presented by various *L. johnsonii* strains, including inhibition of different pathogens in the chick gut, alleviation of diabetes symptoms, reduction of serum cholesterol levels, immunostimulation and adherence to intestinal epithelial cells [24,26-29].

Due to increased interest in *L. johnsonii*, various molecular tools have been used for the precise differentiation of *L. johnsonii* from other members of the *Lactobacillus acidophilus* cluster, particularly the closely related species *Lactobacillus gasseri*[30-33]. The fact that different strains display different characteristics highlights the need to develop tools for their accurate discrimination as well. Various methods have been recently used to type *L. johnsonii* strains, such as pulsed field gel electrophoresis, amplified fragment length polymorphism, enterobacterial repetitive intergenic consensus PCR and repetitive extragenic palindromic PCR [20,21,33,]. These typing methods differ in their discriminatory power, rapidity, complexity, cost, reliability and reproducibility.

In this study we used simple sequence repeats (SSR), also termed variable number tandem repeats (VNTR). SSR loci presents inherently high mutation rate [34], which makes them an appropriate tool for strain typing in many bacterial species [35-37].

Another bacterial typing method based on sequence variations is multiple locus sequence typing (MLST) [38], mainly of housekeeping genes, providing an indication of relatively distant evolutionary processes [39]. Similarly, conserved hypothetical genes can provide an additional source of sequence variation [40]. This cluster of genes with unknown function is predicted to be present in the genomes of all members of a particular species.

In this study *L. johnsonii* was identified and isolated from a selected narrow spectrum of the fecal LAB population originated from various animal hosts. The genetic relationships among *L. johnsonii* strains were inferred based on variation at selected sets of SSR loci and MLST of conserved hypothetical genes. Our findings suggest specificity of *L. johnsonii* strains to their hosts.

## Results

### Isolation of *L. johnsonii* from various animal hosts and characterization of their selected fecal LAB populations

A large survey for *L. johnsonii* isolation was performed, where 104 fecal samples originating in six host taxonomic classes were tested. The isolation procedure of *L. johnsonii* relied on few methods: identifying *L. johnsonii* within a narrow spectrum of fecal LAB populations using terminal restriction fragment length polymorphism (tRFLP) analysis and isolation of suspected *L. johnsonii* colonies based on their morphology followed by species-specific PCR amplification of *23 S rDNA* and *16 S rDNA* sequencing.

**Figure 1:**
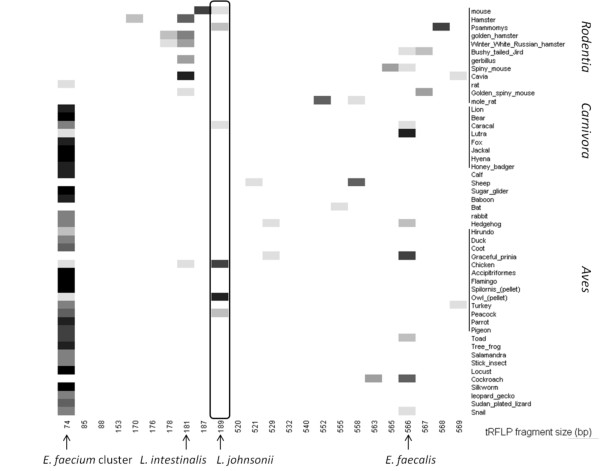
**Relative abundances of tRFLP fragments of selected fecal LAB species from 50 diverse hosts.** Selected Lactic acid bacteria (LAB) of representative host individuals were grown on m-*Enterococcus* agar, where four major bacterial species were identified. Analysis was performed using R software. Shading represents relative abundance, divided into eight levels, with darker shading indicating higher abundance.

The tRFLP patterns of the fecal LAB populations grown on the selective medium m*Enterococcus* agar (based on [8]) generally presented four major peaks representing the *Enterococcus faecium* species cluster, *Lactobacillus intestinalisL. johnsonii*, and *Enterococcus faecalis*. We compared the relative abundance of these bacterial species obtained from 50 different animal hosts from a wide variety of taxonomic groups (Figure 1). No correlation was found between the relative abundance of any of the four bacterial species and either geographical location or nutritional habits of the host (data not shown). However, a correlation was found between the taxonomy classification of the host and the relative abundance of two bacterial species: *L. intestinalis* and the *E. faecium* species cluster. The *E. faecium* cluster was highly abundant in most samples (Figure 1), but appeared at significantly lower levels in samples originated from hosts belonging to the *Rodentia* (F = 32.3975, *p* < 0.0001). In contrast, *L. intestinalis* was relatively rare in general, but was significantly more abundant in samples originated from hosts belonging to the *Rodentia* (F = 6.5525, *p* = 0.0133). In addition, the presence of *L. intestinalis* was correlated with the absence of *E. faecium* cluster and *vice versa*. In contrast to the *E. faecium* cluster and *L. intestinalis*, the appearance of *L. johnsonii* and *E. faecalis* did not correlate with any taxonomic group. In a second step we studied *L. johnsonii* to the strain level. All together 39 strains were isolated from few tested animal hosts (Table 1Additional file 1: Origin of samples collected from 104 animal hosts) and further genetically characterized.

**Table 1:** ** *L. johnsonii* strains isolated from feces samples originated in various animals**

**Isolate name**	**Origin**
LJ_313, LJ_320	Domestic chicken line (laying hen)
LJ_323	Domestic chicken line (local line)
LJ_363, LJ_365, LJ_368, LJ_c1-2, LJ_c2-6, LJ_c2-8, LJ_c4-6, LJ_c5-1	Domestic chicken line (broiler, Ross strain) from two different farms
LJ_c3-6, LJ_c3-4	Domestic chicken line (broiler, Cobb strain)
LJ_ch1, LJ_332, LJ_337	Domestic chicken line (White Rock broiler)
LJ_c6-5, LJ_352, LJ_353	Turkey
LJ_16, LJ_31, LJ_32, LJ_36	Mice (line C57BL/6 J), from two different breeders
LJ_5, LJ_10, LJ_16-1, LJ_18	Mice (line BALB/C)
LJ_11-2	Winter white Russian hamster
LJ_12-5	Golden hamster
LJ_15-8	Rat
LJ_56, LJ_9-7	*Psammomys* (a type of rat)
LJ_184, LJ_188	Owl (*Bubo*; sample was obtained from pellet sample)
LJ_252	Caracal
LJ_4-4	Silkworm
LJ_Mika1	Dog
LJ_380	Calf
LJ_440	Peacock
NCC 533, NCC 1646, NCC 1657, NCC 1669, NCC 1717, NCC 1741	Human (Nestle Research Center)
NCC 1627	Unknown source (Nestle Research Center)
NCC 1703	Cheese (Nestle Research Center)

### Polymorphism at SSR loci along the *L. johnsonii* genome

#### In silico genome-wide screen of L. johnsonii NCC 533 revealed thousands of SSR tracts that were evenly distributed and highly abundant along the genome

Eleven loci with the largest number of repeats were chosen for genetic characterization of *L. johnsonii* (Table 2), having motif sizes ranging from 1 to 480 bp. Ten SSR loci were located in coding regions and one mononucleotide repeat (MNR) locus was located in a noncoding region. Multiple alleles were found at the studied SSR loci among 47 isolates from various hosts, including eight additional strains mainly from humans (generous gift from Nestle Company, Table 1), revealing a high level of polymorphism among *L. johnsonii* strains (Table 2). Two strategies were used to identify the polymorphism: sizing for the SSR loci, and sequencing for the MNR locus. Most SSR loci did not amplify any product (a null allele) in some of the isolates (Table 2). Variation at the MNR locus was observed only in the repeated tract, while the flanking sequences were conserved among isolates. All SSR loci presented 2 to 10 alleles with corresponding diversity indices ranging from 0.28 to 0.76.

**Table 2:** **Number of alleles and diversity index values at the studied 14 loci among *L. johnsonii* isolates**

**Locus**	**Core motif size (bp) and no. of repeats**^**a,b**^	**Gene product**	**No. of alleles or ST**^**c,d**^	**Diversity index**
**SSR loci**				
LJ480	(480)_3_	Hypothetical protein	5	0.47
LJ90	(90)_9_	Hypothetical protein	7	0.56
LJ66	(66)_7_	Hypothetical protein	5	0.50
LJ27	(27)_6_	Hypothetical protein	10	0.76
LJ18	(18)_3_	Hypothetical protein	2	0.28
LJ12	(12)_4_	Signal recognition particle receptor FtsY	7	0.72
LJ9	(9)_3_	Phosphoenolpyruvate-dependent sugar phosphotransferase system EIIC	3	0.66
LJ6	(6)_7_	Putative tyrosine-protein kinase	6	0.74
LJ6_1	(6)_3_	Cell-wall associated serine proteinase	3	0.29
LJ3	(3)_5_	Hypothetical protein	4	0.64
LJ_mono	(1)_11_	Noncoding	5	0.44
**MLST**	Sequence length^b^ (bp)			
LJ0017^e^	1113	‘Conserved hypothetical’ gene	23	
LJ0648	522	‘Conserved hypothetical’ gene	24	
LJ1632	286	‘Conserved hypothetical’ gene	10	

^a^ Subscript numbers are numbers of motif repeats. SSR loci have non-perfect repeats except for loci LJ3 and LJ_mono.

^b^ Based on the genome sequence of *L. johnsonii* NCC 533.

^c^ Allele: number of repeat variant at SSR; ST: number of sequence types at ‘Conserved hypothetical’ genes.

^d^ No. of alleles or ST: MLST genes and SSR loci, except for the locus LJ3, included a null allele.

^e^ Isolates: LJ_352, LJ_353, LJ_363, LJ_365, LJ_ch1, LJ_c2-8, LJ_c5-1, LJc_3-4 and LJ_c6-5 had a deletion of 903 bp.

### Sequence variation at conserved hypothetical genes

Three conserved hypothetical genes were chosen for MLST (Table 2). Most isolates gave the expected product size, except for nine isolates which had a deletion of 903 bp in the LJ0017 gene. The *Psammomys* isolate (LJ_56) did not amplify any product in any of the genes. Sequence variation among isolates was rather high (12.3%), yielding 236 SNPs out of 1922 bp sequenced in the three genes (Table 2). This variation ranged from 10 to 24 sequence types at a gene, including null alleles, indicating rather high variation among *L. johnsonii* strains.

### Phylogenetic analyses

The variation data at SSR loci and conserved hypothetical genes were used in two separate analyses to infer the genetic relationships among *L. johnsonii* isolates.

**Figure 2:**
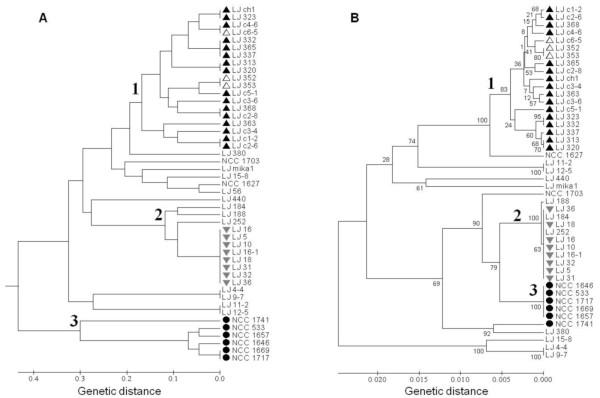
**Genetic relationships among *L. johnsonii* isolates.** Dendograms are based on variation data of: (**A**) 47 isolates at 11 SSR loci based on 57 polymorphic points (11 loci times the number of alleles in each locus); (**B**) sequence of 46 isolates at three conserved hypothetical genes. Both dendrograms were constructed by UPGMA cluster analysis. Samples from: chickens - ▲, turkeys - △, humans - • and mice - ▽ are indicated. All the isolation sources of the tested *L. johnsonii* strains are indicated at Table 1.

SSR analysis: The phylogenetic analysis divided the 47 *L. johnsonii* isolates into 29 different SSR types, revealing high discrimination. The resulting dendrogram presented three main clusters (Figure 2A), one composed of chicken and turkey isolates, the second of human isolates and the third of identical mouse isolates together with strains isolated from the caracal feces and the owl pellet (LJ_184, LJ_188, LJ_16 and LJ_252). Note that the owl pellet isolates might be related to the mouse isolates, as it might have originated from the owl's prey (a mouse), rather than from the owl's upper GIT. The isolates from other diverse origins were spread out along the dendrogram. Among them, isolates from *Psammomys* (LJ_9-7) and silkworm (LJ_4-4), two unrelated host species, are undistinguished according to the typing results. This might be due to their common isolation location, thus additional sampling should clarify the phylogeny clustering of *L. johnsonii* isolates from these two host species. The genetic distances within strains from each of the three groups were significantly low (average genetic distance of 0.25 ± 0.11, 0.27 ± 0.25 and 0.11 ± 0.12 for chicken, human and mouse clusters, respectively) compared to the high genetic distances observed between isolates from the tested group and the remaining isolates (average genetic distance of 0.65 ± 0.18, 0.87 ± 0.10 and 0.64 ± 0.12 for chicken, human and mouse clusters, respectively).

MLST analysis: phylogenetic analysis of the sequences at the three conserved hypothetical genes separated the 46 typable *L. johnsonii* isolates into 28 sequence types (Figure 2B). Three clear clusters were obtained, paralleling the SSR analysis, with the exception of strain NCC 1741.

In general, the two genetic analyses similarly separated *L. johnsonii* isolates into three groups (Figure 2A, 2B). The clusters included strains with a common isolation host: various lines of chicken and turkey, humans, and laboratory mouse lines, while the isolates originating from other diverse sources were dispersed along the dendrograms.

## Discussion

The gut is a habitat for complex bacterial populations, composed of a large variety of bacterial species. Here we concentrated in *L. johnsonii*, a potentially probiotic bacterial species that is of major interest to the pharmaceutical and food industries as it includes several known probiotic strains [25,28,29]. We successfully identified and isolated 39 *L. johnsonii* strains from fecal-bacterial populations of few host species.

Strain typing of these isolates together with six additional strains of human origin revealed high levels of genetic variation among the *L. johnsonii* strains. Both SSR and MLST analyses were found to be effective for typing, providing high-resolution discrimination also among isolates originated in the same animal species. The genetic relationships among the strains inferred by the two analyses were similar, clearly dividing the *L. johnsonii* strains into three clusters. Each cluster consisted of strains from different diverse hosts, *i.e.,* chickens, humans or mice (Figure 2). These consistent results, obtained by different typing methods, suggest far phylogenetic separation among *L. johnsonii* isolates presenting host specificity. Such association of particular *L. johnsonii* strains with the host taxonomy could arise as a result of co-evolution of the host and its GIT microbiota [2,41-43]. Interestingly, host driven evolution was observed in another lactobacilli species, *L. reuteri*[44]. According to the recently suggested "hologenome theory" [45], the host and its symbiont microbiota (together defined as the "holobiont") are one unit of selection in evolution. Indeed, previous analysis of the *L. johnsonii* genome showed the absence of genes required for several metabolic pathways [29] emphasizing the high dependence of *L. johnsonii* on its host and further supports the concept that *L. johnsonii* and its host are one evolutionary unit of selection. Since chickens, humans and mice are distinct genetic species divided during evolution, *L. johnsonii* strains associated with them may be evolutionary separated as part of the distinct holobionts.

In addition, analysis conducted on the tRFLP results of 50 host individuals suggest an association of *L. intestinalis* and *E. faecium* cluster with host taxonomic groups (Figure 1), and further support co-evolution of the host and its intestinal bacteria. The *E. faecium* species cluster was relatively rare in hosts belonging to the *Rodentia* taxonomic order, and alternatively, *L. intestinalis* was found to be more frequent within that group. These observations may indicate possible competition or a similar function of these two bacteria in the same niche, each within its appropriate microenvironment. Environmental factors, such as diet, are highly important in shaping the host gut's microbiota composition [4-6,46]. However, in our study, no correlation was found between the presence of each of the four bacterial species tested and the hosts' food consumption (herbivore, omnivore and carnivore) or geographical location.

## Conclusions

*L. johnsonii* strain typing conducted here support host specificity of *L. johnsonii* only at the strain level. tRFLP analysis of a narrow spectrum of fecal LAB populations demonstrated host specificity of *L. intestinalis* and the *E. faecium* cluster at the species level of bacteria. Both observations suggest co-evolution of the bacteria, either at the species or the strain level, with distinct animal species.

The identified bacterial host specificity may be further applied to utilization of health-promoting specific strains based on the bacterium and the host’s genetics, as part of the personalized medicine approach.

## Methods

### Isolation procedure and growth conditions

A total of 104 samples were collected from a wide variety of animal hosts, originated in 58 animal species. Samples were collected in Israel during a 1.5 year period (January 2009 - June 2010). 102 samples were feces samples, and 2 were bird pellets, *i.e* the materials regurgitated by the birds (see Additional file 1: Origin of samples collected from 104 animal hosts). Each sample, obtained from individual host, was treated and analyzed separately. Samples were kept at 4°C in 0.1 M sodium phosphate buffer pH 7 until arrival to the lab (up to 4 h from the collection time) and processed immediately. 0.1 M sodium phosphate buffer pH 7 was added to a final concentration of 10% (w/v), to equally normalize the growth of fecal bacteria from all samples (see below) according the feces weight. Samples were homogenized by vigorous vortexing, followed by centrifugation at 1500 × *g*, at 4°C for 5 min. The supernatant containing the bacterial suspension was transferred to a clean tube. A 100 μ l aliquot of bacterial suspension was spread on either MRS agar (de Man, Rogosa, Sharpe; Oxoid, UK) or DIFCO m-*Enterococcus* agar plates (BD, Maryland, USA), and grown under both aerobic and anaerobic conditions at 37°C for 48 h. m*Enterococcus* agar was used to isolate *L. johnsonii* based on our previous study [8].

Total DNA was extracted from samples of the bacterial populations grown on the anaerobically incubated m*Enterococcus* agar plates and terminal restriction fragment length polymorphism (tRFLP) was performed, in order to assess the presence of *L. johnsonii* within the total bacterial population that grew on the plate. tRFLP was conducted only for plates that presented massive bacterial growth, estimated at few dozen colonies and more (plates from 62 samples). These samples belong to hosts from six taxonomic classes, in which *Mammalia* (34 samples) and *Aves* (18 samples) were the most abundant. The mammalian hosts belonged to eight different orders, most from *Rodentia* (15 samples) and *Carnivora* (9 samples). Totally, the 62 samples belong to 50 different animal species.

To isolate *L. johnsonii*, aerobically and anaerobically incubated m*Enterococcus* and MRS agar plates were screened for *L. johnsonii* presence, where primary selection was according to slight differences in colony morphology of different species (in comparison to known *L. Johnsonii*) and based on tRFLP results for the 62 samples. Colonies suspected of being *L. johnsonii* were picked for PCR amplification with species-specific primers designed to the *23 S rDNA* (see section Locus and primer selection). Final verification was achieved by *16 S rDNA* sequencing [GenBank: JN 012220 – JN 012227 for *16 S rDNA* sequences of LJ56, LJ313, LJ363, LJ380, LJc1-2, LJc3-4, LJc3-6 and LJmika1, respectively. The *16 S rDNA* sequences of the other *L. johnsonii* isolates are similar to the sequence of LJ16, GenBank: JF923644]. *16 S rDNA* sequences of colonies with slightly different morphologies were indeed proven not to be *L. johnsonii*.

Pure *L. johnsonii* cultures were grown in MRS broth (de Man, Rogosa, Sharpe; Oxoid, UK) overnight at 37°C, freeze-dried and kept at −20°C in the presence of trehalose and maltodextrin, as previously described [47].

### DNA extraction

Cells were harvested from either a loop full of fecal-bacterial population grown on m*Enterococcus* agar plates or pure overnight culture of *L. johnsonii* (200 μl) grown in MRS broth that was centrifuged at 12,000 × *g* for 1 min. Cells were suspended in 1 ml of 70% ethanol by vigorous vortexing, 33 μl of 3 M sodium acetate (pH 5.2) was added and the samples were incubated at −80°C for 20 min, followed by centrifugation at 12,000 × *g* for 15 min. The supernatant was decanted and the pellet was dissolved in 30 μl of 0.1 × Tris-EDTA buffer (TE). The crude DNA was diluted 10-fold and stored at −20°C.

### tRFLP of fecal-bacterial population

*16 S rDNA* of the fecal-bacterial population was amplified in a total volume of 50 μl using 27 F-FAM fluorophore-labeled primer and 1492R primer [48] together with 10 μl of 1:10-diluted crude DNA, at an annealing temperature of 60°C (see section PCR and Additional file 2: Primers and their annealing temperatures (Tm)). The PCR products were purified by ethanol precipitation and dissolved in 20 μl ddH_2_O. A 1-μg aliquot of the purified PCR product was digested with 20 U *Msp1* restriction enzyme (New England Biolabs) in a total volume of 20 μl for 2 h 15 min at 37°C followed by enzyme inactivation at 65°C for 20 min. A 50-ng aliquot of the digested DNA was loaded into an ABI 3130 genetic analyzer together with 9 μl formamide and 0.5 μl GeneScan 1200 LIZ size standard (Applied Biosystems, California, USA) for size determination. The results were analyzed using GeneMapper 4.0 software (Applied Biosystems).

The species identification of an isolated bacterial colony was performed by terminal restriction fragment analysis followed by *16 S rDNA* sequencing and by *in silico* t-RFLP analysis for verification ( http://insilico.ehu.es/T-RFLP/, [49]).

The GenBank nucleotide accession numbers of the identified species representing the tRFLP peaks of 74, 181, 189 and 566 bp (Figure 1, Additional file 3: tRFLP patterns of a selected fecal LAB populations obtained from three representative animal hosts) are: *Enterococcus faecium* species cluster [GenBank: JF923641, JF923642], *Lactobacillus intestinalis* [GenBank: JF923643], *L. johnsonii* [GenBank: JF923644], and *Enterococcus faecalis* [GenBank: JF923645].

### Screening of the genome for SSR distribution

The complete genomic sequence of *L. johnsonii* NCC 533, obtained from the NCBI database, was screened for perfect SSR (i.e., exact-repeat motifs) using the “SSR” computer program [37,50], and for non-perfect SSR (NP-SSR, i.e. non-exact repeat motifs) using the “ATR Hunter” computer program ( http://bioinfo.cs.technion.ac.il/atrhunter/ATRHunter.htm[51]). Perfect SSR included mononucleotide repeats (MNR) with longer than 5-bp repeats, and large SSR with motif size ≥3 bp repeated more than twice. NP-SSR included only SSR with motif size ≥3 bp and minimal similarity between repeats of more than 70%.

### Locus and primer selection

SSR loci: Eleven loci (Additional file 2: Primers and their annealing temperatures (Tm)) were chosen for the study, including ten SSR loci and one MNR locus. These regions exhibited no similarity to phage or prophage sequences. Unique primers were designed to generate PCR products of 120 to 1650 bp using the Gene Runner software (version 3.05; Hastings Software Inc.). Each locus was tested for uniqueness in the *L. johnsonii* genome by using NCBI BLAST ( http://www.ncbi.nlm.nih.gov/sutils/genom_table.cgi).

Species-specific primers: *L. johnsonii*-specific primers were designed based on the *23 S rDNA* sequences of a variety of lactobacilli available at the NCBI database. The forward primer was designed such that the last nucleotide at the 3’ end of the primer was unique to *L. johnsonii*. The reverse primer was designed based on a previously designed *L. johnsonii*-specific probe [52]. Species-specific PCR amplification (Tm = 51°C, Additional file 2: Primers and their annealing temperatures (Tm)) was performed directly on the colonies of the suspected *L. johnsonii* isolates.

Conserved hypothetical genes: Three conserved hypothetical genes were chosen for the MLST from the JCVI CMR database ( http://cmr.jcvi.org/cgi-bin/CMR/CmrHomePage.cgi) based on the genome sequence of *L. johnsonii* NCC 533. Gene choice was based on two criteria: (i) presence in other *L. johnsonii* strains, and (ii) a high number of single nucleotide polymorphisms (SNPs) compared to the sequence of *L. johnsonii* ATCC 32000 in the NCBI database. Unique primers were designed to generate PCR products of 400 to 1200 bp (Additional file 2: Primers and their annealing temperatures (Tm)). Due to the non-amplification of products in a few strains, additional primer sets were designed for each of the genes (LJ0017_new, LJ_0648_new and LJ_1632_new) based on the sequences obtained for the rest of the isolates.

### PCR

Each PCR mixture contained 0.2 mM deoxynucleoside triphosphates, 0.4 μM forward and reverse primers, 0.02 U/μl of *Taq* polymerase (SuperNova, JMR Holding, Kent, England), 1× reaction buffer (containing 1.5 mM MgCl_2_) and 5 μl of 1:10-diluted crude DNA in a total volume of 25 μl. The reactions were carried out in a Veriti 96-well thermal cycler (Applied Biosystems, California, USA) as follows: 95°C for 3 min; 30 cycles of 30 s at 95°C, 30 s at the annealing temperature (Tm, Additional file 2: Primers and their annealing temperatures (Tm)), and 90 s at 72°C; 10 min at 72°C, and cooling to 12°C. PCR products were verified by gel (1.2%) electrophoresis and observed by UV fluorescence.

### DNA sizing

Size determination of SSR amplification products with motif lengths of 66 bp, 90 bp and 480 bp was performed by 2% agarose gel electrophoresis. Sizing of the other seven SSR loci was performed by capillary electrophoresis on an ABI 3130 genetic analyzer, using fluorophore-labeled primers. The amplification products were loaded into the genetic analyzer together with 9 μl formamide and 0.5 μl GeneScan 500 LIZ size standard (Applied Biosystems). The results were analyzed with GeneMapper 4.0 software (Applied Biosystems).

### DNA sequencing

PCR amplification products were purified using a QIAquick PCR purification kit (Qiagen, Hilden, Germany). Purified DNA (20–50 ng) was sequenced on both strands using a BigDye terminator v1.1 cycle sequencing kit (Applied Biosystems) and loaded into the ABI 3130 genetic analyzer. Results were analyzed with SeqScape 2.5 software (Applied Biosystems) and DNA sequencing analysis 5.2 software (Applied Biosystems).

GenBank numbers of nucleotide sequences for genes LJ_0017, LJ_0648 and LJ_1632: JN012103 – JN 012141, JN 012142 - JN 012180 and JN 012181 - JN 012219 respectively.

### Data and statistical analyses

tRFLP: The relative abundance of each tRFLP peak was calculated as the peak area divided by the total area summed over all peaks in a sample. A statistical analysis was performed for each of the four main tRFLP peaks (74 bp, 181 bp, 189 bp and 566 bp) separately. M-ANOVA (JMP 8.0) was performed based on the relative abundance of each tested peak in each sample to compare its presence among the 50 tested samples under three parameters (geographical location, taxonomy and food classification). The software R was used to present the relative abundances of the tRFLP patterns, split into eight levels.

Sequence comparison: The obtained *16 S rDNA* sequences were compared to all available sequences using the NCBI BLAST algorithm for species identification. The analysis of the sequence variation data was performed on the combined sequences of the three conserved hypothetical genes for each of the 46 strains. One strain (LJ_56) did not give any amplification product and was therefore excluded from the MLST analysis. Multiple sequence alignments were performed using CLUSTALW software [53]. The alignment files were converted to MEGA format and used to evaluate genetic relationships among the strains by the unweighted pair group method with arithmetic mean (UPGMA) (MEGA 4.0 [54]).

Allele analysis: A nonparametric analysis of allelic variation was used for all 47 *L. johnsonii* isolates at SSR loci, with alleles referring to SSR size alleles. For the MNR locus, alleles referred to the MNR size, similar to the SSR loci, as no sequence variation was obtained in the flanking regions of the MNR. An additional allele was counted where there was no amplification product. The data for all genotypes were scored as present (“1”) or absent (“0”) for each allele at a specific locus. Diversity index was calculated as 1 - ∑P^2^_*ij*_, where P_*ij*_ is the frequency of the *j*th allele at the *i*th locus. Genetic relationships were inferred among strains based on the variation data. SAS software was used to calculate the Nei coefficient of association and to generate the corresponding matrix (SAS system for Windows, version 9.02; SAS Institute, Inc., Cary, NC). The matrix was used to create dendograms based on the UPGMA using MEGA 4.0 software [54]. Bootstrap confidence values were based on 1,000 simulated dendrograms.

## Authors' contributions

KB, YD, HS, YK conceived and designed the study. KB, VM and MJ carried out the experiments. KB and YD analyzed results. KB, YD and YK drafted the manuscript. All authors read and approved the final manuscript.

## Supplementary Material

Additional file 1 Origin of samples collected from 104 animal hosts. Click here for file

Additional file 2 Primers and their annealing temperatures. Click here for file

Additional file 3 **tRFLP patterns of selected fecal LAB populations obtained from three representative animal hosts.** Bacteria were grown on m-*Enterococcus* agar. Fluorescent-labeled DNA fragments were analyzed by ABI 3130 genetic analyzer. The size of specific fragments is indicated in bp. The owl sample is a pellet sample. Click here for file
