# Supplementary figures and images for: Indication for Co-evolution of Lactobacillus johnsonii with its hosts
Source: BMC Microbiol. 2012 Jul 25;12:149. doi: 10.1186/1471-2180-12-149 (PMC3503616; doi:10.1186/1471-2180-12-149)

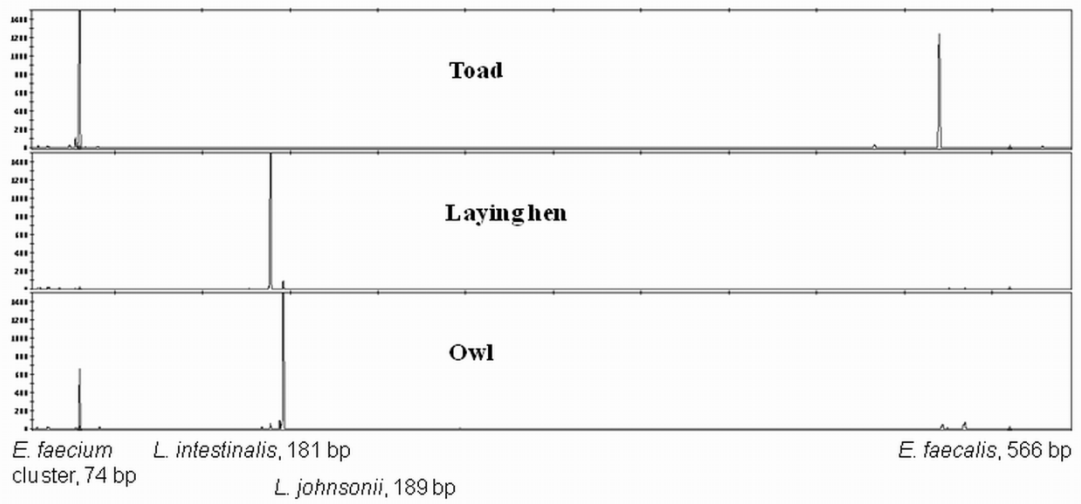

Supplement: Additional file 1 — Origin of samples collected from 104 animal hosts. [file 1471-2180-12-149-S1.pdf]
