# Supplementary material for: Indication for Co-evolution of Lactobacillus johnsonii with its hosts
Source: BMC Microbiol. 2012 Jul 25;12:149. doi: 10.1186/1471-2180-12-149 (PMC3503616; doi:10.1186/1471-2180-12-149)
Supplement: Additional file 2 — Primers and their annealing temperatures. [file 1471-2180-12-149-S2.pdf]

Origin	Sample source	Sampling location[1]	Number of samples containing *L. johnsonii* / Total number of samples
Dog	Feces	Domestic animal	1/2
Psammomys	Feces	Haifa zoo[2]/ Oranim animal house	1/2
Mice	Feces	Technion animal house	7/10
Owl	pellet	Haifa zoo	1/1
Caracal	Feces	Haifa zoo	1/1
Silkworm, winter white Russian hamster, Golden hamster, Rat	Feces	Oranim animal house	1/1
House chicken breeds	Feces	Hayogev farms	12/14
Turkey	Feces	Hayogev farms	2/2
Calf	Feces	Haifa zoo[2]/ Hayogev farms/ Ramat Yohanan frams	1/3
Peacock	Feces	Haifa zoo	1/1
Sheep, Camel, Goat, Honey badger, Toad, Turtle, Tiger, Golden jackal, Hyena, Leopard, Bear, Lutra, Baboon, Sugar glider, Lion, Horse, Flamingo, Chinchilla, Hystrix, Gecko, Dove, Parrot, Pigeon, Human infant, Tree frog, Salamandra, Hirundo, Stick insect, Leopard gecko, Mole rat, Golden spiny mouse, Gerbillus, Bushy-tailed jird, Spiny mouse, Cavia, Sudan-plated lizard, Stellion, Graceful prinia, Gold fish, Snail, Blattaria, Pycnonotus, Coot, Duck, Locust	Feces	Haifa zoo/ Oranim animal house/ Ramat Yohanan frams/ Domestic animal	0/1 each
Circaetus	pellet	Haifa zoo	0/1
Cow, Hamster, Bat, Fox, Hedgehog	Feces	Haifa zoo/ Ramat Yohanan frams/ Hayogev farms/ Domestic animal	0/2 each
Falcon	Feces and pellet	Haifa zoo	0/2
Accipitriformes, Rabbit	feces	Haifa zoo/ Oranim animal house/ Domestic animal	0/3

[1] All locations are in Israel

[2] *L. johnsonii* was isolated from that sample
