# Supplementary material for: Indication for Co-evolution of Lactobacillus johnsonii with its hosts
Source: BMC Microbiol. 2012 Jul 25;12:149. doi: 10.1186/1471-2180-12-149 (PMC3503616; doi:10.1186/1471-2180-12-149)
Supplement: Additional file 3 — tRFLP patterns of selected fecal LAB populations obtained from three representative animal hosts. Bacteria were grown on m-Enterococcus agar. Fluorescent-labeled DNA fragments were analyzed by ABI 3130 genetic analyzer. The size of specific fragments is indicated in bp. The owl sample is a pellet sample. [file 1471-2180-12-149-S3.pdf]

SSR locus/ Gene name	Position in genome (bp)[a]	Primer	Dye	Sequence (5'-3')	T_m (°C)
16S rDNA		27F[b]	FAM	AGAGTTTGATCMTGGCTCAG	60
		1492R[b]	-	TACGGYTACCTTGTTACGACTT	
		Middle[c]	-	CGTAGGTGGCAAGCGTTGT	
23S rDNA		F	-	GTGAGAGCCCCGTAC	51
		R[d]	-	CTACCACGCATATAATATA	
**SSR**					
LJ480	1746596	F	-	CATCTGGGTAAGTTACTGTA	54
		R	-	CTGCTGAAACTAAGAATGC	
LJ90	1724927	F	-	TTAATGGTTATACTACCGATCA	54
		R	-	GAGAATGCCTATATCCGTTG	
LJ66	1554620	F	-	TTTATGGTTACTTATCTCCG	53
		R	-	AACAGCAGAACTCAAGCAG	
LJ27	1800840	F	VIC	CTGCTTCTACTTTACCAGATTC	55
		R	-	ACATTTATACAAGTCATTTCTCC	
LJ18	1239444	F	PET	CTTAGATGGAGTTACAGGCT	63
		R	-	TACTGCTCATAATACTACCCC	
LJ12	1389441	F	FAM	GATTCAGTTTCTTCGGGCTT	53
		R	-	GAAATCGCTCTTTGGTCATA	
LJ9	1529353	F	FAM	CCAGAACCATAAGTTAAAGG	56
		R	-	CCAACTGTTTTAATGACTCAC	
LJ6	923115	F	VIC	TTAGCTGGTGAAATGGATGG	53
		R	-	GCTAACCCTAATGATGATTC	
LJ6_1	1963728	F	NED	GTGCGTTTACTTGTGAGTCT	58
		R	-	CCAAGAATTGAAGCAGGCAA	
LJ3	654914	F	FAM	GATGATAAGAAACATCGCAC	54
		R	-	GCATCAATATCTCCACCGTT	
LJ_mono	58766	F	-	GAGAGACAGGCATAAAAGAT	56
		R	-	TGATACCTTAACAACCTCCT	

**'Conserved Hypothetical' Genes**[e]					
LJ0017	21517	F	-	GTGGAGTTGGTTATGTAGC	53
		R	-	TAAGGACACTAACAAGCAAG	
LJ0648	1714704	F	-	ATTGCGGATGCACTAGATA	53
		R	-	CATAATGCCAATCTTTGAACTG	
LJ1632	1508966	F	-	CGTTGAGTTAAGTAAAGAG	52
		R	-	GATTGCTGTTCTTGATAAGC	
LJ0017_new	21517	F	-	CATTTTCTGAAAGTTTTTATCGA	58
		R	-	TACCTCCAAAAGAAAGCAGC	
LJ0648_new	1714704	F	-	TAATGCCAATCTTTGAACTG	50
		R	-	GGATGCACTAGATAAAGA	
LJ1632_new	1508966	F	-	ACTGATGGTAAGCTTAAAAGCA	58
		R	-	GAATTACTGGAAGATGCAAA	

[a] Based on the genome sequence of *L. johnsonii* NCC 533.

[b] Based on Sakamoto et al. [48].

[c] Used for sequencing reaction only.

[d] Based on Park and Itoh [52].

[e] Locus tags.
